# Low bone mineral density predicts the formation of new syndesmophytes in patients with axial spondyloarthritis

**DOI:** 10.1186/s13075-018-1731-8

**Published:** 2018-10-16

**Authors:** Hyoung Rae Kim, Yeon Sik Hong, Sung-Hwan Park, Ji Hyeon Ju, Kwi Young Kang

**Affiliations:** 1 Division of Rheumatology, Department of Internal Medicine, College of Medicine, The Catholic University of Korea, Seoul, South Korea; 2 Division of Rheumatology, Department of Internal Medicine, Incheon St. Mary’s Hospital, College of Medicine, The Catholic University of Korea, #56, Dongsu-Ro, Bupyung-Gu, Incheon, South Korea

**Keywords:** Axial spondyloarthritis, Ankylosing spondylitis, Bone mineral density, Syndesmophyte

## Abstract

**Background:**

This study aimed to investigate whether the presence of low bone mineral density (BMD) in patients with axial spondyloarthritis (axSpA) predicts formation of new syndesmophytes over 2 years.

**Methods:**

One hundred and nineteen patients fulfilling the imaging arm of the Assessment of SpondyloArthritis International Society axSpA criteria were enrolled. All patients were under 50 years of age. The modified Stoke Ankylosing Spondylitis Spinal Score (mSASSS) was assessed by two trained readers blinded to the patients’ data. BMD (lumbar spine, femoral neck or total hip) at baseline was assessed using dual-energy absorptiometry. Low BMD was defined as *Z* score ≤ − 2.0. Spinal radiographic progression was defined as worsening of the mSASSS by ≥ 2 points over 2 years. Logistic regression analyses were performed to identify predictors associated with development of new syndesmophytes and spinal radiographic progression.

**Results:**

At baseline, 19 (16%) patients had low BMD. New syndesmophytes had developed in 22 (21%) patients at 2-year follow-up. New syndesmophyte formation after 2 years occurred more in patients with low BMD than in those with normal BMD (*p* = 0.047). In the multivariable analysis, current smoking, existing syndesmophytes and low BMD at baseline were associated with spinal radiographic progression (OR (95% CI) 3.0 (1.1, 7.7), 4.6 (1.8, 11.8) and 3.6 (1.2, 11.2), respectively). The presence of syndesmophytes at baseline and low BMD were predictors of new syndesmophytes over the following 2 years (OR (95% CI) 5.5 (2.0, 15.2) and 3.6 (1.1, 11.8), respectively).

**Conclusions:**

Low BMD and existing syndesmophytes at baseline were independently associated with the development of new syndesmophytes in young axSpA patients.

## Background

Axial spondyloarthritis (axSpA) is a chronic inflammatory disease that predominantly affects the sacroiliac joints and the spine. axSpA includes the subtypes ankylosing spondylitis (AS) and non-radiographic axSpA (nr-axSpA). These separate entities are discriminated by the structural damage to the sacroiliac joints visible on conventional X-ray images [1].

For most patients with SpA, the burden of disease results from a combination of inflammation and structural bone damage [2]. Radiographic damage in the spine presents as syndesmophyte formation leading to bridging of the intervertebral spaces. Structural damage not only affects patients by causing disability and permanent loss of function, but also has secondary effects, with ankylosis changing the balance of loads and forces on the skeletal system, leading to muscle stiffness and accelerated degenerative spine disease [3]. As structural damage contributes to impairment of spinal mobility and function, the retardation of spinal radiographic progression should be an important treatment goal [4].

Spinal progression varies widely among axSpA patients, and previous studies examined predictors influencing the heterogeneous formation of syndesmophytes within these patients. The strongest predictor of radiographic spinal progression is the presence of syndesmophytes at baseline [5, 6]. Furthermore, increased levels of acute phase reactants and smoking are independent predictors of radiographic spinal progression in early axSpA patients [6].

In a recent study, persisting high disease activity according to the Ankylosing Spondylitis Disease Activity Score (ASDAS) was found to be associated with accelerated radiographic spinal progression in early axSpA patients [7]. This advocates the early use of anti-inflammatory treatment in patients with early and active disease, in the hope that decreasing the disease activity will also slow down the radiographic progression. Identification of the predictors of spinal progression at baseline is important for clinical decision-making on aggressive anti-inflammatory treatment.

Chronic inflammation of the spine leads not only to new bone formation in axial joints and vertebral spaces, but also to bone resorption leading to osteoporosis, which is increased in axSpA [8]. It has been established that the generalised bone loss may be due to systemic inflammation and disease activity [9], and as disease activity in AS contributes to the rate of bone loss, osteoporosis is considered to be a manifestation of the disease itself, rather than a comorbidity [10].

Low bone mass in axSpA is a result of increased bone resorption through differentiation and activation of osteoclasts caused by inflammation. Therefore, bone mass changes reflect the severity of persistent inflammation, rather than a time-specific inflammatory state. Trabecular bone loss has been clearly and repeatedly demonstrated in the spines of patients with axSpA [11]. In a recent study, trabecular bone microarchitecture was found to be associated with spinal structural damage, as well as systemic inflammatory markers [12]. Furthermore, inflammation on spinal MRI is related to low bone mass in patients with nr-axSpA [13]. These studies show the site-specific relationship between low bone mass and inflammation in the spine. Earlier studies also reported the association between low bone mass in AS and spinal structural damage [14–16]; however, despite the identification of this relationship, it is not yet known whether the presence of low bone mass can independently predict radiographic spinal progression in axSpA patients.

The aims of the present study were therefore to evaluate the association between low bone mass and the formation of new syndesmophytes, and to investigate whether low bone mass independently predicts radiographic progression in axSpA patients.

## Methods

### Study population

Between August 2013 and December 2015, consecutive axSpA patients from Incheon Saint Mary’s Hospital (Incheon, Korea) were recruited to this study. All enrolled patients fulfilled the imaging arm of the Assessment of SpondyloArthritis International Society (ASAS) axSpA criteria [17]. To exclude the effects of age, patients aged 50 years or older were excluded. Further exclusion criteria included patients with thyroid or parathyroid disorders, the presence of chronic renal or liver disease, cancer, coeliac disease or concurrent rheumatoid arthritis and the use of corticosteroids.

Bone mineral density (BMD) using dual-energy absorptiometry (DXA) and lateral radiographs of the cervical and lumbar spine were assessed at the time of enrolment, and demographic data were collected at the time of BMD assessment. All participants provided written informed consent according to the Declaration of Helsinki, and the study was approved by the ethics committee at Incheon Saint Mary’s Hospital (study number OC16OISI0138).

### Clinical data

Disease-related data and disease activity scores were collected at baseline. Clinical data included the time after symptom onset, the presence of HLA B27, family history and peripheral arthritis. A 44-joint count has been proposed to measure peripheral joint involvement, with this including the sternoclavicular joints, acromioclavicular joints, shoulders, elbows, wrists, knees and ankles, and the MCP, MTP and PIP joints of the hands [18]. Measures of disease activity were collected using the Bath Ankylosing Spondylitis Disease Activity Index (BASDAI) [19]. The ASDAS was calculated as described in a previous study [20]. The Bath Ankylosing Spondylitis Functional Index (BASFI) [21] was also recorded, and the erythrocyte sedimentation rate (ESR) and C-reactive protein (CRP) were measured. The use of medications such as non-steroidal anti-inflammatory drugs (NSAIDs), sulfasalazine, tumour necrosis factor (TNF) inhibitors, calcium, bisphosphonate and vitamin D was recorded, with patients who had taken treatment agents for periods of 1 year or longer being considered sustained users.

### Radiographic scoring

For all patients, conventional radiographs of the spine were obtained at baseline and at 2-year follow-up. Lateral views of the cervical and lumbar spine were scored according to the modified Stoke Ankylosing Spondylitis Spinal Score (mSASSS) [22]. The mSASSS was scored by two trained experts who were blinded to the patients’ demographic and clinical data and orders. In the mSASSS, a lateral view of the anterior parts of the cervical and lumbar spine is scored for squaring and/or erosion and/or sclerosis (1 point), syndesmophytes (2 points) and bridging syndesmophytes (3 points). As determination of cervical spine squaring may be unreliable, this element was not scored on the radiographs [23]. The total scores ranged from 0 to 72. Significant 'spinal radiographic progression' was defined as an increase in the mSASSS of ≥ 2 units over 2 years [7]. In the present analysis, the formation of new syndesmophytes at individual vertebral levels was of interest. Therefore, a ‘new syndesmophyte’ was defined as the formation of a syndesmophyte (mSASSS 2 points) or a bridge (mSASSS 3 points) at a vertebral level that was previously uninvolved or with only signs of squaring, erosion or sclerosis at baseline (mSASSS 0 or 1 point).

Two investigators independently scored the baseline and 2-year follow-up radiographs for each patient. To quantify the reliability of the radiographic scoring, the intraclass correlation coefficients (ICCs) for status scores (one time point) and change scores were calculated. There were few discrepancies between the two independent trained readers, but when they did occur the two investigators reached a consensus.

### BMD assessment

BMD of the lumbar spine and left hip was assessed using DXA (Lunar Prodigy densitometer, Madison, WI, USA) at the baseline enrolment. All measurements were taken by experienced operators using the same machine and standardised procedures for participant positioning. BMD was measured at the lumbar spine using an anteroposterior projection at L1–L4, and at the left hip from the femoral neck and total proximal femur, and was expressed as the number of grams of bone mineral per square centimetre (g/cm^2^) and calculated as *Z* scores using the manufacturer’s reference. For patients under 50 years of age, a *Z* score ≤ − 2.0 standard deviations (SDs) relative to the age-matched mean is considered to be below the expected range [24]; therefore, low BMD was defined as *Z* score ≤ − 2.0.

### Statistical analysis

Continuous data are expressed as the mean ± SD, and categorical data are expressed as percentages. Clinical variables were compared using independent *t* tests, and the chi-squared test was used to compare categorical variables between axSpA patients with and without a new syndesmophyte. The numbers of syndesmophytes at baseline and 2-year follow-up were compared using a paired *t* test.

Between-reader agreement in the determination of numbers of syndesmophytes at baseline and 2-year follow-up was estimated by the ICC. Agreement between the two readers regarding the presence of syndesmophytes was very good both at baseline (ICC 0.967 (95% CI 0.953–0.977)) and at 2 years (ICC 0.970 (95% CI 0.957–0.979)). Agreement over the change in the number of syndesmophytes was also good (ICC 0.739 (95% CI 0.625–0.818)).

Univariable and multivariable logistic regression analyses were used to identify predictors associated with the formation of new syndesmophytes and spinal radiographic progression over the 2-year follow-up period. Odds ratios (ORs) with 95% CIs were calculated. Variables identified in the univariate analysis (*p* <  0.05) were entered into a backward stepwise multiple logistic regression model.

*p* ≤ 0.05 was considered statistically significant. Statistical analysis was performed with IBM SPSS Statistics version 18.

## Results

A total of 217 patients with axSpA were enrolled at baseline. All patients were aged between 20 and 50 years. For 126 of the 217 patients, radiographs in the spine were available at baseline and at 2-year follow-up. Four patients with total ankylosis in the cervical and lumbar spine at enrolment were excluded from the analysis. One patient with stomach cancer, one patient with concurrent rheumatoid arthritis and one patient with chronic hepatitis B were also excluded. Thus, 119 patients with definite axSpA were included in this analysis. Baseline characteristics of the total group, as well as for the groups stratified according to the formation of new syndesmophytes, are presented in Table 1. In total, 34 (29%) patients had syndesmophytes at baseline. Ninety (76%) patients had radiographic sacroiliitis fulfilling the modified New York criteria for the classification of AS [25], and 19 (16%) patients had low BMD.

**Table 1:** Baseline patient characteristics

Variable	Total patients (*n* = 119)	New syndesmophytes at 2 years		*p* value
		No (*n* = 97)	Yes (*n* = 22)	
Age (years)	35 ± 9	34 ± 10	38 ± 8	0.075
Male sex	91 (77)	74 (76)	17 (77)	1.000
BMI (kg/m^2^)	23.2 ± 3.4	22.9 ± 3.2	24.4 ± 4.1	0.072
Current smoking	36 (30)	25 (26)	11 (50)	0.038
Alcohol ≥ 3 units/day	3 (3)	3 (3)	0 (0)	1.000
Symptom duration (years)	8.6 ± 7.6	7.9 ± 7.3	11.6 ± 8.0	0.035
Family history of axSpA	12 (10)	12 (12)	0 (0)	0.120
HLA B27-positive	110 (92)	90 (93)	20 (91)	0.671
Peripheral arthritis	27 (23)	22 (23)	5 (23)	1.000
Radiographic sacroiliitis	90 (76)	70 (72)	20 (91)	0.097
BASDAI score (range 0–10)	3.9 ± 2.1	3.9 ± 2.0	3.7 ± 2.7	0.683
BASFI score	1.7 ± 2.1	1.7 ± 2.2	1.8 ± 1.8	0.722
ESR (mm/h)	23 ± 20	23 ± 20	23 ± 19	0.960
CRP (mg/L)	9.1 ± 14.9	8.9 ± 15.2	10.2 ± 14.1	0.703
ASDAS-ESR	2.6 ± 1.1	2.6 ± 1.1	2.5 ± 1.3	0.707
ASDAS-CRP	2.2 ± 1.3	2.2 ± 1.2	2.2 ± 1.5	0.883
mSASSS	7.6 ± 14.3	5.7 ± 13.0	15.8 ± 17.1	0.015
Number of syndesmophytes	2.3 ± 4.8	1.6 ± 4.3	5.0 ± 6.0	0.021
Presence of syndesmophytes	34 (29)	21 (22)	13 (59)	0.001
Patients on NSAIDs	106 (89)	86 (89)	20 (91)	1.000
Patients on sulfasalazine	42 (35)	34 (35)	8 (36)	1.000
Patients on TNF inhibitors	36 (30)	27 (28)	9 (41)	0.303
Patients on bisphosphonate	2 (2)	2 (2)	0 (0)	1.000
Patients on calcium	17 (14)	11 (11)	6 (27)	0.085
Patients on vitamin D	18 (15)	12 (12)	6 (27)	0.099
BMD (g/cm^2^)				
Lumbar spine	1.16 ± 0.17	1.17 ± 0.16	1.13 ± 0.22	0.949
Femoral neck	0.94 ± 0.14	0.94 ± 0.14	0.92 ± 0.16	0.401
Total hip	0.98 ± 0.15	0.99 ± 0.15	0.95 ± 0.15	0.338
Low BMD (*Z* score ≤ −2.0)				
Any site	19 (16)	12 (12)	7 (32)	0.047
Lumbar spine	15 (13)	10 (10)	5 (23)	0.150
Femoral neck	3 (3)	1 (1)	2 (12)	0.070
Total hip	5 (4)	3 (3)	2 (9)	0.233

Data presented as *n* (%) or mean ± standard deviation

*BMI* body mass index, *axSpA* axial spondyloarthritis, *BASDAI* Bath Ankylosing Spondylitis Disease Activity Index, *BASFI* Bath Ankylosing Spondylitis Functional Index, *ESR* erythrocyte sedimentation rate, *CRP* C-reactive protein, *ASDAS* Ankylosing Spondylitis Disease Activity Score, *mSASSS* Modified Stoke Ankylosing Spondylitis Spinal Score, *NSAID* non-steroidal anti-inflammatory drug, *TNF* tumour necrosis factor, *BMD* bone mineral density

New syndesmophytes had developed in 22 (21%) patients at the 2-year follow-up. The patient group with new syndesmophytes had a higher percentage of current smokers at baseline and patients with a longer symptom duration than the patient group without new syndesmophytes (*p* = 0.038 and *p* = 0.035, respectively). Patients with new syndesmophytes had a higher frequency of syndesmophytes at baseline and a higher baseline mSASSS score than those who did not develop new syndesmophytes (*p* = 0.001 and *p* = 0.015, respectively). Low BMD in any site (lumbar spine, femoral neck or total hip) at baseline was more frequent in patients with new syndesmophytes (*p* = 0.047). There were no significant differences in disease activity scores, inflammatory markers and treatment agents.

Patients with low BMD at baseline showed higher levels of ESR and CRP (*p* = 0.019 and *p* = 0.022, respectively), as presented in Table 2. Patients with low BMD received more calcium agent and vitamin D treatments.

**Table 2:** Baseline patient characteristics in relation to low BMD at baseline

Variable	Low BMD at baseline		*p* value
	No (*n* = 100)	Yes (*n* = 19)	
Age (years)	35 ± 9	32 ± 11	0.245
Male sex	75 (75)	16 (84)	0.558
BMI (kg/m^2^)	23.1 ± 3.2	23.5 ± 4.2	0.671
Current smoking	30 (30)	6 (32)	1.000
Alcohol ≥ 3 units/day	3 (3)	0 (0)	1.000
Symptom duration (years)	8 ± 7	9 ± 8	0.615
Family history of axSpA	10 (10)	2 (11)	1.000
HLA B27-positive	92 (92)	18 (95)	1.000
Peripheral arthritis	24 (24)	3 (16)	0.559
Radiographic sacroiliitis	73 (73)	17 (90)	0.154
BASDAI score (range, 0–10)	3.9 ± 2.2	3.8 ± 2.0	0.849
BASFI score	1.7 ± 2.1	1.9 ± 2.0	0.672
ESR (mm/h)	21 ± 20	32 ± 18	0.019
CRP (mg/L)	7.4 ± 13.4	18.0 ± 19.2	0.032
ASDAS-ESR	2.5 ± 1.2	2.9 ± 1.0	0.169
ASDAS-CRP	2.1 ± 1.3	2.7 ± 1.2	0.051
mSASSS	7 ± 14	11 ± 16	0.215
Number of syndesmophytes	2.1 ± 4.7	3.1 ± 5.6	0.430
Presence of syndesmophytes	28 (28)	6 (32)	0.785
Patients on NSAIDs	88 (88)	18 (95)	0.690
Patients on sulfasalazine	33 (33)	9 (47)	0.296
Patients on TNF inhibitors	28 (28)	8 (42)	0.227
Patients on bisphosphonate	0 (0)	2 (11)	0.024
Patients on calcium	10 (10)	7 (37)	0.006
Patients on vitamin D	10 (10)	8 (42)	0.002

Data presented as *n* (%) or mean ± standard deviation

*BMD* bone mineral density, *BMI* body mass index, *axSpA* axial spondyloarthritis, *BASDAI* Bath Ankylosing Spondylitis Disease Activity Index, *BASFI* Bath Ankylosing Spondylitis Functional Index, *ESR* erythrocyte sedimentation rate, *CRP* C-reactive protein, *ASDAS* Ankylosing Spondylitis Disease Activity Score, *mSASSS* modified Stoke Ankylosing Spondylitis Spinal Score, *NSAID* non-steroidal anti-inflammatory drug, *TNF* tumour necrosis factor

**Fig. 1:**
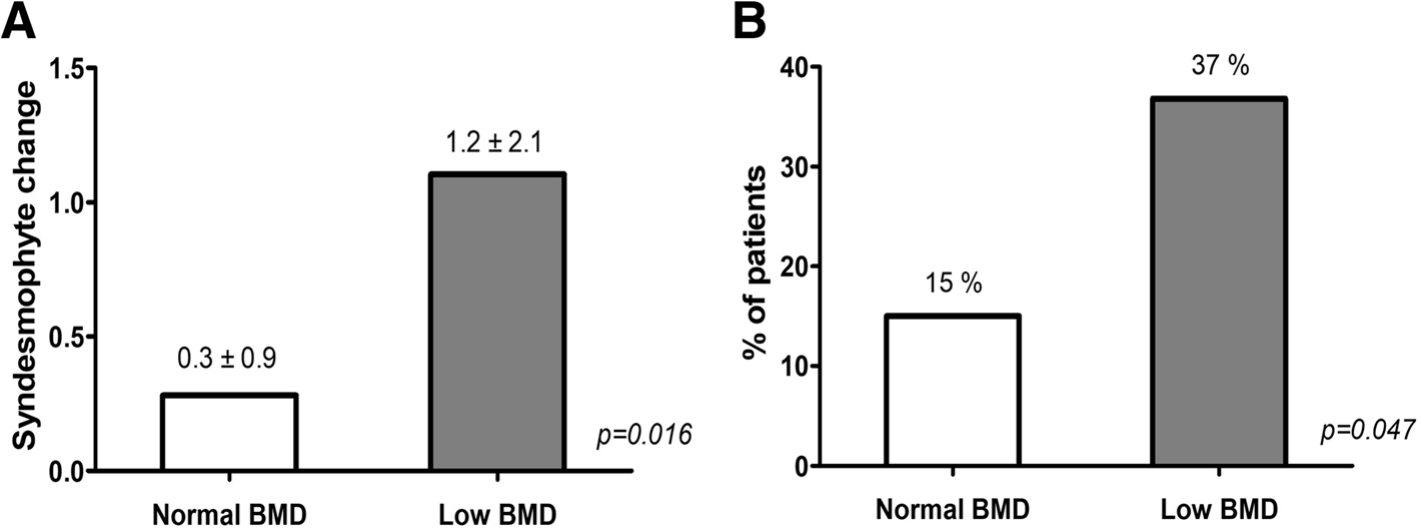
Absolute change in syndesmophyte numbers (**a**) and proportions of patients with new syndesmophytes (**b**) according to presence of low bone mineral density (BMD) at baseline

Table 3 presents the mean number of syndesmophytes at baseline and 2-year follow-up for the total group and the groups with or without low BMD at baseline. The number of syndesmophytes significantly increased in the period from baseline to 2 years in the total patient group (0.4 over 2 years) and both subgroups dichotomised according to low BMD. In the group of patients without low BMD at baseline, the mean increase was 0.3 over the 2 years, while in those with low BMD at baseline it was 1.2 (Fig. 1a). New syndesmophytes after 2 years occurred in 15% of patients without low BMD and in 37% of patients with low BMD (*p* = 0.047; Fig. 1b).

**Table 3:** Number of syndesmophytes at baseline and after 2 years for the total group and groups stratified for the presence or absence of low BMD

Group	Baseline syndesmophytes	2-year syndesmophytes	*p* value
Total patients (*n* = 119)	2.3 ± 4.8	2.7 ± 5.4	< 0.001
Normal BMD (*n* = 100)	2.1 ± 4.7	2.4 ± 5.0	< 0.001
Low BMD (*n* = 19)	3.0 ± 5.6	4.2 ± 7.4	< 0.001

Values presented as mean ± standard deviation

*BMD* bone mineral density

**Table 4:** Univariable and multivariable analysis of significant progression of mSASSS over 2 years

Variable	Univariable analysis			Multivariable analysis^a^		
	OR	95% CI	*p* value	OR	95% CI	*p* value
Male sex	0.5	0.2–1.5	0.223			
Age (years)	1.1	1.0–1.1	0.019			
Symptom duration (years)	1.1	1.0–1.1	0.064			
BMI (kg/m^2^)			0.817			
< 18.5	1.0	0.2–5.3	0.964			
18.5–22.9	1.0 (reference)					
23.0–24.9	1.4	0.5–4.0	0.487			
≥ 25.0	1.5	0.6–4.2	0.408			
Current smoking	4.1	1.7–9.6	0.001	3.0	1.1–7.7	0.025
HLA B27-positive	1.3	0.3–6.7	0.743			
Peripheral arthritis	0.5	0.2–1.6	0.269			
Radiographic sacroiliitis	4.1	1.2–14.7	0.029			
Presence of syndesmophytes	5.7	2.4–13.8	< 0.001	4.6	1.8–11.9	0.002
BASFI score, per 1 point	1.0	0.8–1.2	0.678			
BASDAI score ≥ 4	0.9	0.7–1.1	0.328			
ASDAS-CRP			0.313			
Low (< 1.3)	1.0 (Reference)					
Moderate (< 2.1)	1.2	0.4–3.6	0.769			
High (≤ 3.5)	0.3	0.2–1.3	0.135			
Very high (> 3.5)	0.9	0.2–3.2	0.839			
Increased ESR (≥ 20 mm/h)	1.1	0.5–2.4	0.842			
Increased CRP (≥ 5 mg/L)	1.4	0.6–3.3	0.431			
Patients on NSAIDs	0.7	0.2–3.1	0.716			
Patients on sulfasalazine	0.7	0.3–1.9	0.530			
Patients on TNF inhibitors	1.6	0.7–3.7	0.298			
Patients on calcium	3.0	0.8–11.3	0.097			
Patients on vitamin D	2.1	0.4–10.1	0.336			
Low BMD, any site (*Z* score ≤ − 2.0)	3.0	1.1–8.3	0.033	3.6	1.2–11.2	0.028

*mSASSS* Modified Stoke Ankylosing Spondylitis Spinal Score, *OR* odds ratio, *CI* confidence interval, *BMI* body mass index, *BASFI* Bath Ankylosing Spondylitis Functional Index, *BASDAI* Bath Ankylosing Spondylitis Disease Activity Index, *ASDAS* Ankylosing Spondylitis Disease Activity Score, *CRP* C-reactive protein, *ESR* erythrocyte sedimentation rate, *NSAID* non-steroidal anti-inflammatory drug, *TNF* tumour necrosis factor, *BMD* bone mineral density

^a^Adjusted for age, smoking, radiographic sacroiliitis, presence of syndesmophytes and presence of low BMD at any site at baseline

Among the total of 119 patients, 27% (32) showed spinal radiographic progression (change of mSASSS ≥ 2 points) over 2 years. In the univariable logistic regression analysis, age (OR 1.1), current smoking (OR 4.1), radiographic sacroiliitis (OR 4.1), presence of syndesmophytes (OR 5.7) and low BMD (OR 3.0) at baseline were associated with spinal radiographic progression (Table 4). In the multivariable analysis, current smoking, presence of syndesmophytes and low BMD at baseline were independently associated with significant spinal progression (OR 3.0 (95% CI 1.1–7.7), OR 4.6 (95% CI 1.8–11.9) and OR 3.6 (95% CI 1.2–11.2), respectively). In the univariable logistic regression analysis to identify predictors of the formation of new syndesmophytes over 2 years (Table 5), symptom duration (OR 1.1), current smoking (OR 2.9), presence of syndesmophytes (OR 5.2) and low BMD (OR 3.3) at baseline were statistically significant factors and were included in the subsequent multivariable logistic regression analysis. This identified the presence of baseline syndesmophytes and low BMD at any site (OR 5.5 (95% CI 2.0–15.2) and OR 3.6 (95% CI 1.1–11.8), respectively) as significant predictors of new syndesmophytes.

**Table 5:** Univariable and multivariable analysis of the formation of new syndesmophytes over 2 years

Variable	Univariable analysis			Multivariable analysis^a^		
	OR	95% CI	*p* value	OR	95% CI	*p* value
Male sex	1.0	0.3–2.9	0.922			
Age (years)	1.0	1.0–1.1	0.078			
Symptom duration (years)	1.1	1.0–1.1	0.040			
BMI (kg/m^2^)			0.838			
< 18.5	1.7	0.3–9.8	0.567			
18.5–22.9	1.0 (reference)					
23.0–24.9	1.5	0.4–4.9	0.534			
≥ 25.0	1.6	0.5–5.2	0.403			
Current smoking	2.9	1.1–7.5	0.029			
HLA B27-positive	0.8	0.2–4.0	0.765			
Peripheral arthritis	1.0	0.3–3.0	0.996			
Radiographic sacroiliitis	3.9	0.8–17.6	0.082			
Presence of syndesmophytes	5.2	2.0–13.9	0.001	5.5	2.0–15.2	0.001
BASFI score, per 1 point	1.0	0.8–1.3	0.720			
BASDAI score ≥ 4	1.0	0.4–2.6	0.965			
ASDAS-CRP			0.632			
Low (< 1.3)	1.0 (reference)					
Moderate (< 2.1)	1.1	0.3–3.8	0.900			
High (≤ 3.5)	0.5	0.2–1.7	0.278			
Very high (> 3.5)	0.3	0.2–3.3	0.688			
Increased ESR (≥ 20 mm/h)	1.3	0.5–3.2	0.630			
Increased CRP (≥ 5 mg/L)	1.6	0.6–4.2	0.320			
Patients on NSAIDs	1.3	0.3–6.2	0.761			
Patients on sulfasalazine	1.1	0.4–2.8	0.907			
Patients on TNF inhibitors	1.8	0.7–4.7	0.232			
Patients on calcium	2.9	0.9–9.0	0.062			
Patients on vitamin D	2.7	0.9–8.1	0.086			
Low BMD, any site (*Z* score ≤ −2.0)	3.3	1.1–9.8	0.030	3.6	1.1–11.8	0.031

*OR* odds ratio, *CI* confidence interval, *BMI* body mass index, *BASFI* Bath Ankylosing Spondylitis Functional Index, *BASDAI* Bath Ankylosing Spondylitis Disease Activity Index, *ASDAS* Ankylosing Spondylitis Disease Activity Score, *CRP* C-reactive protein, *ESR* erythrocyte sedimentation rate, *NSAID* non-steroidal anti-inflammatory drug, *TNF* tumour necrosis factor, *BMD* bone mineral density

^a^Adjusted for symptom duration, presence of syndesmophytes, smoking and presence of low BMD at any site at baseline

## Discussion

In this longitudinal observational study, we investigated the formation of new syndesmophytes in the spines of patients with axSpA over a period of 2 years, and identified predictors of new syndesmophyte formation and spinal radiographic progression. About 20% of the patients developed a new syndesmophyte over 2 years. The presence of a syndesmophyte at baseline and low BMD were predictors of the formation of new syndesmophytes and significant mSASSS progression.

Abnormal bone metabolism in axSpA is characterised by pathological new bone formation in the cortical zone of the vertebrae and the loss of trabecular bone from the centres of the vertebral bodies. Osteoproliferation leads to syndesmophytes, while the loss of trabecular bone leads to low BMD [26].

This study is the first to demonstrate that low BMD predicts radiographic progression in axSpA. The main determinants of low BMD in axSpA patients are systemic inflammation and bone-specific inflammation [27]. The inflammatory process is associated with altered systemic bone remodelling, increased bone resorption and impaired bone formation resulting from the effects of inflammatory mediators on the differentiation and activity of osteoclasts and osteoblasts. Proinflammatory cytokines can influence osteoclastogenesis and osteoblastic activity [28]. Thus, the presence of low BMD in axSpA is considered to be a result of altered bone remodelling caused by persistent inflammation. In the present study, serum levels of ESR correlated with BMD values of the lumbar spine, femoral neck and total hip, and *Z* scores of the femoral neck and total hip. Additionally, CRP levels correlated with the *Z* score at the lumbar spine and femoral neck (data not shown).

Bone loss resulting from chronic inflammation and the associated changes in bone microarchitecture have been proposed as a potential driving mechanism for the ankylosing process [29]. The inflammatory process induces bone loss, which affects the microarchitecture in the trabecular bone, thereby leading to instability. Reduced bone strength triggers a stabilising anabolic effort that results in bone formation. Trabecular and cortical compartments appear to have different reactions to inflammation; in axSpA, inflammation has a direct effect on the trabecular bone of the vertebrae, but not on the cortical bone [8]. As persistent inflammation in the trabecular bone of the vertebral bodies may prevent the anabolic response from correcting the instability, new bone formation in the cortical bone of the vertebrae may be increased [30]. This would result in the formation of syndesmophytes: compensatory stability for the spine but with a loss of normal mobility [31].

Another explanation is that low BMD may represent the presence of a repairing area that was affected by active inflammation in the past. There is increasing evidence that new bone formation in axSpA is the consequence of previous inflammation in the subchondral bone marrow, with the appearance of granulated repair tissue occurring as a mandatory intermediate step, with this then being followed by new bone formation. It has been proposed that the best current treatment for the prevention of bone formation is the early and effective suppression of bony inflammation [32]. Additionally, if it is possible to detect the presence of repair tissue, the risk of new syndesmophyte formation could also be predicted. The presence of a low BMD means that the repair process may become apparent, to compensate for bone loss resulting from the inflammatory process. Therefore, the presence of low BMD may represent areas with ongoing repair affected by inflammation, such as fatty lesions on MRI.

In the current study, the presence of baseline syndesmophytes in axSpA was found to be the strongest predictor for the formation of new syndesmophytes. This finding is consistent with those of earlier studies. Similar results with respect to spinal radiographic progression have been found in early axSpA patients, as well as in AS patients [5, 33, 34]. However, a substantial proportion of patients with baseline syndesmophytes do not show progression over 2 years. Furthermore, it is arguable whether the presence of baseline syndesmophytes should be considered a true predictor, because the patients already exhibited the features that the model was designed to predict [5].

Smoking is reported to be associated with spinal radiographic progression in early axSpA [6] and the radiographic severity of AS [35], although the exact mechanisms for the influence of smoking on radiographic progression are not known. In the present study, smoking was associated with new syndesmophyte formation only in the univariable analysis, whereas it was independently associated with significant mSASSS progression over 2 years after adjustment for confounding factors. Only the status of smoking at baseline was included in the analysis, and the relatively small number of patients may have influenced the inconsistent results with respect to smoking. The influence of smoking on new syndesmophyte formation should be studied in a larger cohort.

The presence of radiographic sacroiliitis was significantly associated with spinal radiographic progression over 2 years, but this significant association was not present in the multivariable analysis. This finding is consistent with a previous result in early axSpA patients [6]. In the present study, 90% of patients with low BMD had radiographic sacroiliitis. This high proportion of radiographic sacroiliitis in the patients with low BMD could have affected the result. The exact effect of radiographic sacroiliitis on spinal progression should be clarified in a large patient cohort including non-radiographic axSpA patients with low BMD.

Although we did not observe a significant result in this study, systemic inflammatory markers were reported as predictors of radiographic progression in patients with early axSpA in the German Spondyloarthritis Inception Cohort (GESPIC) [6]. This previous study included early axSpA patients with short symptom duration (mean 4.2 years) and low mSASSS (mean 4.25) [6]. In this study, the mean symptom duration and mSASSS were 8.6 years and 7.6 points. These differences suggest that our study patients had more severe and longstanding symptoms and were advanced patients. In a prospective observation of a cohort with longstanding AS, inflammatory markers did not emerge as independent predictors, as per the results of the present study [5]. These discordant results could possibly be explained by the differences in disease duration and structural damage severity. Furthermore, we only analysed the associations between baseline ESR and CRP measurements and new syndesmophyte formation, not time-averaged inflammatory markers. Baseline inflammatory markers may be less reflective of the status of persistent systemic inflammation than time-averaged values. Therefore, our results for the predictive role of systemic inflammation should be interpreted with caution.

Inflammation plays a key role in bone loss in axSpA, and anti-inflammatory drugs are expected to have a beneficial effect on bone through both the increased mobility related to pain relief and the direct effects on bone [28]. The best treatment for the prevention of bone formation/progression is currently the early and effective suppression of bony inflammation [32]. Our results suggest that successful anti-inflammatory treatment reduces inflammation and allows the bone metabolism to normalise, thereby taking away the compensatory anabolic response that leads to new bone formation in the cortical bone of the spine. Although we did not find a beneficial effect of NSAIDs or TNF inhibitors in this 2-year follow-up study, recent long-term follow-up data show that TNF inhibitors suppress radiographic spinal progression [36, 37]. Thus, if patients are treated for a longer time with a TNF inhibitor (preventing new occurrences of the sequence of inflammation, repair and new bone formation), or if they are treated early in the course of their disease, such a treatment seems to be effective in retarding the process of new bone formation [32]. Taken together, active anti-inflammatory treatment is crucial for the prevention of spinal ankylosis in young axSpA patients, especially those with low BMD.

This study has some limitations. First, the number of axSpA patients was relatively small; therefore, the regression analysis could be underpowered. In our patients, those with low BMD received more calcium agents and vitamin D treatments, and these agents could have affected the bone metabolism. Although calcium and vitamin D intake were not significant in the regression analysis, their effects on spinal progression need to be clarified in a large cohort including more patients with low BMD. The BMD in axSpA can be affected by the presence of syndesmophytes or other structural lesions such as an ankylosed posterior arch and periosteal bone formation. Therefore, the BMD of the lumbar spine in patients with syndesmophytes could have been overestimated. Lastly, it is known that there is an association between serum levels of sex hormones and BMD in AS [38], but these were not measured in this study.

## Conclusions

The presence of low BMD and syndesmophytes at baseline were independently associated with the formation of new syndesmophytes in young axSpA patients. Current smoking, syndesmophytes and low BMD at baseline were also associated with significant mSASSS progression. Effective anti-inflammatory treatment may modify radiographic spinal progression in young axSpA patients with low BMD. Our findings require confirmation in other large cohorts of axSpA patients.

## Abbreviations

AS: Ankylosing spondylitis
ASAS: Assessment of SpondyloArthritis International Society
ASDAS: Ankylosing Spondylitis Disease Activity Score
axSpA: Axial spondyloarthritis
BASDAI: Bath Ankylosing Spondylitis Disease Activity Index
BASFI: Bath Ankylosing Spondylitis Functional Index
BMD: Bone mineral density
CRP: C-reactive protein
DXA: Dual-energy absorptiometry
ESR: Erythrocyte sedimentation rate
ICC: Intraclass correlation coefficient
mSASSS: Modified Stoke Ankylosing Spondylitis Spinal Score
nr-axSpA: Non-radiographic axSpA
NSAID: Non-steroidal anti-inflammatory drug
OR: Odds ratio
SD: Standard deviation
TNF: Tumour necrosis factor
